# Supplementary material for: Dynalign II: common secondary structure prediction for RNA homologs with domain insertions
Source: Nucleic Acids Res. 2014 Nov 21;42(22):13939–48. doi: 10.1093/nar/gku1172 (PMC4267632; doi:10.1093/nar/gku1172)
Supplement: SUPPLEMENTARY DATA [file supp_gku1172_nar-02021-z-2014-File012.zip › manual/Overview/Cleaning_the_Repository.html]

RNAstructure Installation and Overview: Cleaning the Repository


|  |  |  |
| --- | --- | --- |
|  | RNAstructure Installation and Overview Cleaning the Repository | - Contents - Index |
| A special note on cleaning the repository  When the "make realclean" target of the main Makefile is invoked, it calls its "make clean" target to remove object files throughout the repository, then completely cleans out the exe/ directory.  This cleaning removes all files and folders in the exe/ directory that weren't part of the original repository, not just executables.  For this reason, it is recommended that output files, directories, or other necessary files be created outside of the exe/ directory.  Otherwise, they may accidentally get deleted when the repository is cleaned. | | |
| Visit The Mathews Lab RNAstructure Page for updates and latest information. | | |
